# An open-label phase II study of low-dose thalidomide in androgen-independent prostate cancer

**DOI:** 10.1038/sj.bjc.6600817

**Published:** 2003-03-18

**Authors:** M J Drake, W Robson, P Mehta, I Schofield, D E Neal, H Y Leung

**Affiliations:** 1 Department of Surgery, School of Surgical Sciences, The Medical School, University of Newcastle, Newcastle upon Tyne NE2 4HH, UK; 2 Department of Urology, Freeman Hospital, Freeman Road, High Heaton, Newcastle upon Tyne NE7 7DN, UK; 3 Department of Neurology, Newcastle General Hospital, Westgate Road, Newcastle upon Tyne NE74 6BE, UK

**Keywords:** prostate cancer, androgen-independent, thalidomide

## Abstract

The antiangiogenic effects of thalidomide have been assessed in clinical trials in patients with various solid and haematological malignancies. Thalidomide blocks the activity of angiogenic agents including bFGF, VEGF and IL-6. We undertook an open-label study using thalidomide 100 mg once daily for up to 6 months in 20 men with androgen-independent prostate cancer. The mean time of study was 109 days (median 107, range 4–184 days). Patients underwent regular measurement of prostate-specific antigen (PSA), urea and electrolytes, serum bFGF and VEGF. Three men (15%) showed a decline in serum PSA of at least 50%, sustained throughout treatment. Of 16 men treated for at least 2 months, six (37.5%) showed a fall in absolute PSA by a median of 48%. Increasing levels of serum bFGF and VEGF were associated with progressive disease; five of six men who demonstrated a fall in PSA also showed a decline in bFGF and VEGF levels, and three of four men with a rising PSA showed an increase in both growth factors. Adverse effects included constipation, morning drowsiness, dizziness and rash, and resulted in withdrawal from the study by three men. Evidence of peripheral sensory neuropathy was found in nine of 13 men before treatment. In the seven men who completed six months on thalidomide, subclinical evidence of peripheral neuropathy was found in four before treatment, but in all seven at repeat testing. The findings indicate that thalidomide may be an option for patients who have failed other forms of therapy, provided close follow-up is maintained for development of peripheral neuropathy.

Prostate cancer is the second leading cause of cancer deaths in men and curative therapy in the form of radical surgery or radiotherapy is limited to patients with organ-confined disease. Endocrine treatment by androgen ablation is a palliative measure for patients with locally advanced or metastatic prostate cancer, but the response to endocrine therapy is temporary, usually in the range 18–24 months, as castration appears to favour a subpopulation of cells that seem to thrive in the absence of androgens (Logothetis *et al*, 1994). This property is thought to be dependent on the autocrine or paracrine effects of growth factors, acting either directly on the tumour cells, or indirectly on new blood vessel formation (angiogenesis). Many agents have been tested with the aim of improving the outlook in hormone-refractory disease, with clinical response in terms of serum prostate-specific antigen (PSA) levels and tumour mass observed in a minority. No regimen has been found to provide a substantial improvement in survival time and, importantly, many are detrimental to quality of life.

Thalidomide is a sedative, anti-inflammatory and immunosuppressive agent, which has been used or proposed for use in various serious illnesses, including AIDS- and cancer-related cachexia, leprosy and multiple sclerosis. Several clinical trials have employed thalidomide in refractory malignancy with varying degrees of success. *In vitro* data suggests that thalidomide has anti- angiogenic activity, blocking the expression of multiple angiogenic agents (D'Amato *et al*, 1994). Accordingly, an open-label phase II study of thalidomide at a dose of 100 mg daily in androgen-independent prostate cancer was conducted to evaluate its efficacy and tolerability. During the same period, Figg *et al* investigated the use of thalidomide in patients with androgen-independent prostate cancer at higher doses (daily doses of 200 mg up to 1200 mg), with greater overall benefit observed in the low-dose arm.

## MATERIAL AND METHODS

### Patient characteristics

The main inclusion criterion for entry to the trial was a rising serum PSA after initial response to hormonal manipulation therapy. All patients had histological confirmation of prostate adenocarcinoma and were managed at initial diagnosis by primary androgen ablation with LHRH agonist injections or bilateral subcapsular orchidectomy. Androgen-independence was defined as a rising PSA value of at least 20 ng ml^−1^ on two consecutive occasions after the nadir of response to androgen ablation therapy, or a rise of at least 5 ng ml^−1^ if the absolute PSA value was less than 20 ng ml^−1^. Thus all patients had progressive disease on biochemical criteria (Bubley *et al*, 1999). Exclusion criteria included any unstable medical condition, long-term corticosteroid therapy, surgery or radiotherapy in the preceding 28 days, spinal cord compression or severe bone pain requiring immediate treatment. No patient had previously received chemotherapy for their prostate cancer.

### Study design

Thalidomide (Sauramide, Penn Pharmaceuticals, Tredegar, UK) 100 mg was given once daily at bedtime for up to 6 months in an open-label phase II study. All patients continued on prior hormone therapy, except for the discontinuation of antiandrogen medication with a 1 month washout period.

Patients were assessed monthly to detect symptomatic adverse drug reactions. Serum PSA, urea and electrolytes, liver function tests and haematology were determined each month. Samples for measurement of cytokine levels were processed within 1 h and serum stored at −20°C until used for ELISA for bFGF/FGF2 and VEGF, according to the manufacturer's instructions (R&D, UK). Lower urinary tract symptoms were assessed with the ICS male questionnaire and performance status with the Medical Outcomes Study Short Form 36 (SF 36) questionnaire (Ware *et al*, 1993) every 3 months. Nerve conduction studies (NCS) were undertaken at screening and after 6 months, using bilateral lower limb recording of the response amplitude and conduction velocity from two sensory (sural and superficial peroneal) and two motor (common peroneal and posterior tibial) nerves (Johnsen and Fuglsang-Fredericksen, 2000). All of the sensory nerve action potential (SNAP) data were pooled for analysis.

The protocol was approved by the Newcastle and North Tyneside Health Authority Joint Ethics Committee, with written informed consent from each subject.

### Response evaluation

The primary end point of the study was the assessment of changes in PSA based on the intention to treat. Decline in absolute PSA value was defined as a reduction compared with the screening value, which was maintained for at least 4 weeks. Patients showing a decline in serum PSA of at least 50% without clinical evidence of progression were considered to show a PSA response, according to the guidelines of the Prostate-Specific Antigen Working Group (Bubley *et al*, 1999). In addition, PSA velocity was recorded, derived from the gradient of log plots of the PSA against time, prior to and following initiation of thalidomide. Secondary end points were evaluation of toxicity, tolerability and alterations in levels of circulating growth factors.

### Statistical analysis

Paired and unpaired *t*-tests were used for parametric data and the Mann–Whitney *U*-test for nonparametric data, with statistical significance inferred at *P*<0.05.

## RESULTS

A total of 20 men were recruited (Table 1

**Table 1:** Characteristics of recruited patients

**Characteristic**	
Mean age (range)	71.1 (60–83) years
Mean pretreatment PSA (range)	305 (14–3302) ng ml^−1^
Gleason score for histology (range)	7.9 (6–10)
Local stage (T3/T4)	12/8
Bony metastases present	13 (65%)
Concurrent LHRH analogue therapy	14 (70%)
Previous bilateral subcapsular orchidectomy	6 (30%)

) and mean time on study was 109 days (median 107, range 4–184 days). Reasons for withdrawal were clinical progression resulting in bone pain (5) or ureteric obstruction (2), adverse drug effects (3), colonic perforation (1), inability to comply with frequent hospital visits (1) and withdrawal of consent (1) (demographic data given in Table 2

**Table 2:** Characteristics of men failing to complete the study

**Characteristic**	
Time on study; mean (median)	69 (61) days
Mean age (range)	72.5 (60–83)
Mean pretreatment PSA (range)	451 (56–3302) ng ml^−1^
Gleason score for histology (range)	8.4 (7–10)
Local stage (T3/T4)	7/6
Bony metastases present	9 (69%)
Concurrent LHRH analogue therapy	8 (61%)
Previous bilateral subcapsular orchidectomy	5 (38%)

). At screening, the seven men who showed clinical progression on thalidomide did not differ significantly from the study group overall in respect of age, histological grade or performance status, but the PSA was higher (median 322 *vs* 95 ng ml^−1^, *P*=0.06). Subjects were followed up for a median of 13 months after discontinuing thalidomide (mean 12, range 5–16 months). Nine men had died at a median of 2 months following treatment (mean 3.7, range 0–10 months).

### Prostate-specific antigen response

Prostate-specific antigen data are set out in Table 3

**Table 3:** Prostate-specific antigen data

**Patient**	**Screening** **PSA (ng ml^−1^)**	**PSA nadir (ng ml^−1^)**	**% PSA decline**^a^	**Pretrial PSA vel. (ng ml^−1^ month^−1^)**	**Trial PSA vel. (ng ml^−1^ month^−1^)**
1^b^	87	DNF	—	19 (22%)	9 (10%)
2	111	DNF	—	31 (28%)	4 (4%)
3	3302	1256	62	514 (16%)	−2046 (−62%)
4^b^	152	35	77	27 (18%)	−19 (−13%)
5	322	178	45	20 (6%)	−104 (−32%)
6	170	170	0	8 (5%)	−4 (2%)
7^b^	86	65	24	17 (20%)	−14 (−16%)
8^b^	46	DNF	—	5 (11%)	1 (2%)
9	56	50	11	25 (45%)	−6 (−11%)
10	69	NA	—	NA	NA
11^b^	74	DNF	—	8 (11%)	7 (9%)
12	585	399	32	244 (42%)	−132 (−23%)
13	102	DNF	—	15 (15%)	16 (16%)
14^b^	25	DNF	—	13 (52%)	2 (8%)
15	81	DNF	—	8 (10%)	11 (14%)
16	143	140	2	4 (3%)	−20 (−14%)
17	65	NA	—	18 (28%)	NA
18	400	NA	—	133 (33%)	NA
19	400	NA	—	43 (11%)	NA
20^b^	14	7	50	3 (21%)	−5 (−36%)

a Between screening PSA and nadir.

b Patients completing full 6 months on thalidomide. DNF=did not fall. NA=not applicable.

and values for the seven men who completed the full 6 month study period (patient numbers 1, 4, 7, 8, 11, 14 and 20) are illustrated in Figure 1

**Figure 1:**
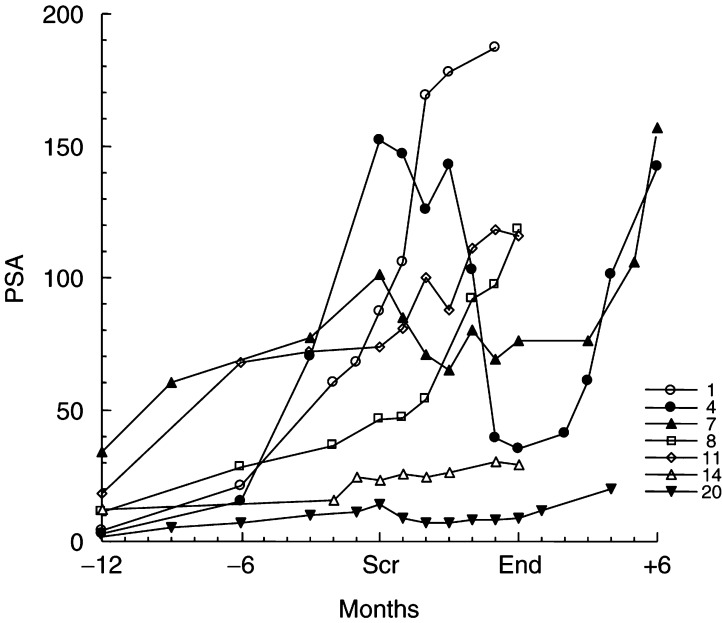
Prostate-specific antigen data for seven men completing 6 months on low-dose thalidomide. Prostate specific antigen values are shown for the study period and the preceding 12 months. For the three men showing a fall in PSA in response to thalidomide (patient numbers 4, 7 and 20, filled icons), values for the period up to 6 months after completion of the study are also illustrated. Scr=screening, ‘End’ refers to completion of the trial.

. Three of these seven (numbers 4, 7 and 20) showed a fall in absolute PSA by a mean of 50% (range 24–77%), the PSA falling over the study period and rising after discontinuation of the drug in each case. The remaining four men did not show a fall in PSA. Prostate-specific agent data were available for a further nine men receiving treatment for at least 2 months. In this group, the PSA fell in three by a mean of 46% (range 32–62%). One patient showed an initial fall of 11% in the first month, but the PSA rose above screening levels thereafter. Four men did not receive thalidomide for 2 months, so their PSA data could not be analysed. Overall, six out of the 16 men receiving thalidomide for at least 2 months (37.5%) showed a fall in their serum PSA levels by a mean of 48% (median 48%). Of these, three (18.8%) showed a fall in PSA of at least 50%, representing 15% of the 20 patients initially recruited with the intention to treat. Absolute PSA level, PSA velocity, performance status, Gleason score and presence of bone metastases did not influence the likelihood of PSA response.

### Serum growth factor levels

**Figure 2:**
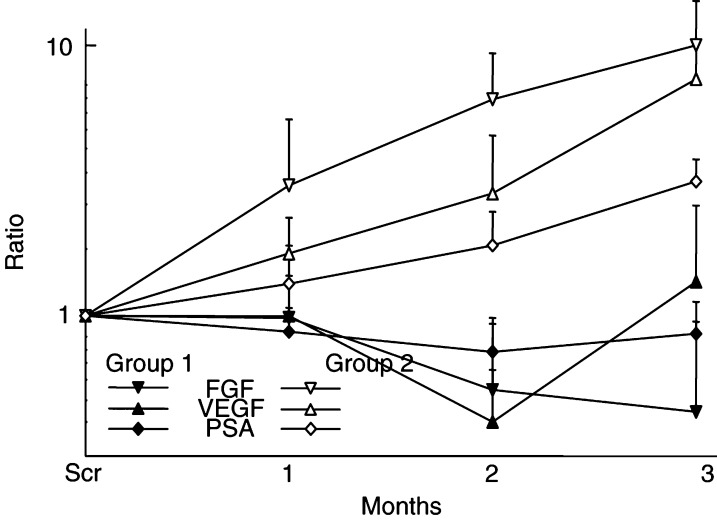
Prostate-specific antigen and growth factor changes. Changes in mean serum PSA, bFGF and VEGF during the first 3 months of taking thalidomide for men showing a decline (‘group 1’, closed icons, *n*=6) or a rise (‘group 2’, open icons, *n*=4) in PSA. Values for each marker are normalised to a value of 1 at screening and plotted on a logarithmic scale. Error bars represent one standard deviation.

Changes in serum bFGF and VEGF over the initial 3-month period were evaluated in 11 patients. Overall, bFGF rose from 2.4±2.6 pg ml^−1^ to 6.3±5.8 (mean±s.d.), and VEGF rose from 262.4±215.4 to 337.5±333.9 pg ml^−1^. Subgroup analysis according to PSA response showed concurrent changes in serum growth factor levels and PSA (Figure 2

). Five out of six men manifesting a fall in PSA (‘group 1’) showed a decline in mean values for both bFGF and VEGF to 0.6±0.93 and 151.5±75.2 pg ml^−1^ at the third month, respectively. A slight rise in serum PSA in the third month in this group coincided with a rise in VEGF, while the bFGF continued to fall. Conversely, three out of four patients with a rising PSA (‘group 2’) showed an increase in levels of both bFGF (22.1±34.3 pg ml^−1^) and VEGF (521.0±353.4 pg ml^−1^). The difference between subgroups after treatment showed statistical significance for bFGF (*P*=0.04) but not VEGF (*P*=0.18). One patient who did not clearly fall into either group in terms of PSA changes showed a rise in bFGF and a fall in VEGF.

### Toxicity

Adverse drug effects are listed in Table 4

**Table 4:** Adverse drug effects

**Adverse effect**	***n***	**Discontinued therapy**
Constipation	9	0
Sedation hangover	3	1
Dizziness	2	1
Transient ischaemic attack	1	1
Clinical peripheral neuropathy	0	0
Dry skin	1	0
Rash	1	0

. Constipation usually responded to dietary advice and mild oral aperients, but one patient with a past history of diverticular disease withdrew from the study on developing peritonitis following perforation of a colonic diverticulum. This man did not have rectal stenosis because of his prostatic adenocarcinoma and constipation associated with thalidomide was possibly a contributory factor. ‘Sedation hangover’, defined as drowsiness for several hours after rising, was reported by three people. One person developed a transient morbiliform rash after taking the drug for several weeks, which affected the forearms and legs and resolved over a 2-week period. Another patient with no past history of cerebrovascular events reported symptoms resembling a transient ischaemic attack 4 days after starting thalidomide. Two patients developed acute urinary retention after 10 and 17 weeks on study and were only able to void following transurethral resection of the prostate.

**Figure 3:**
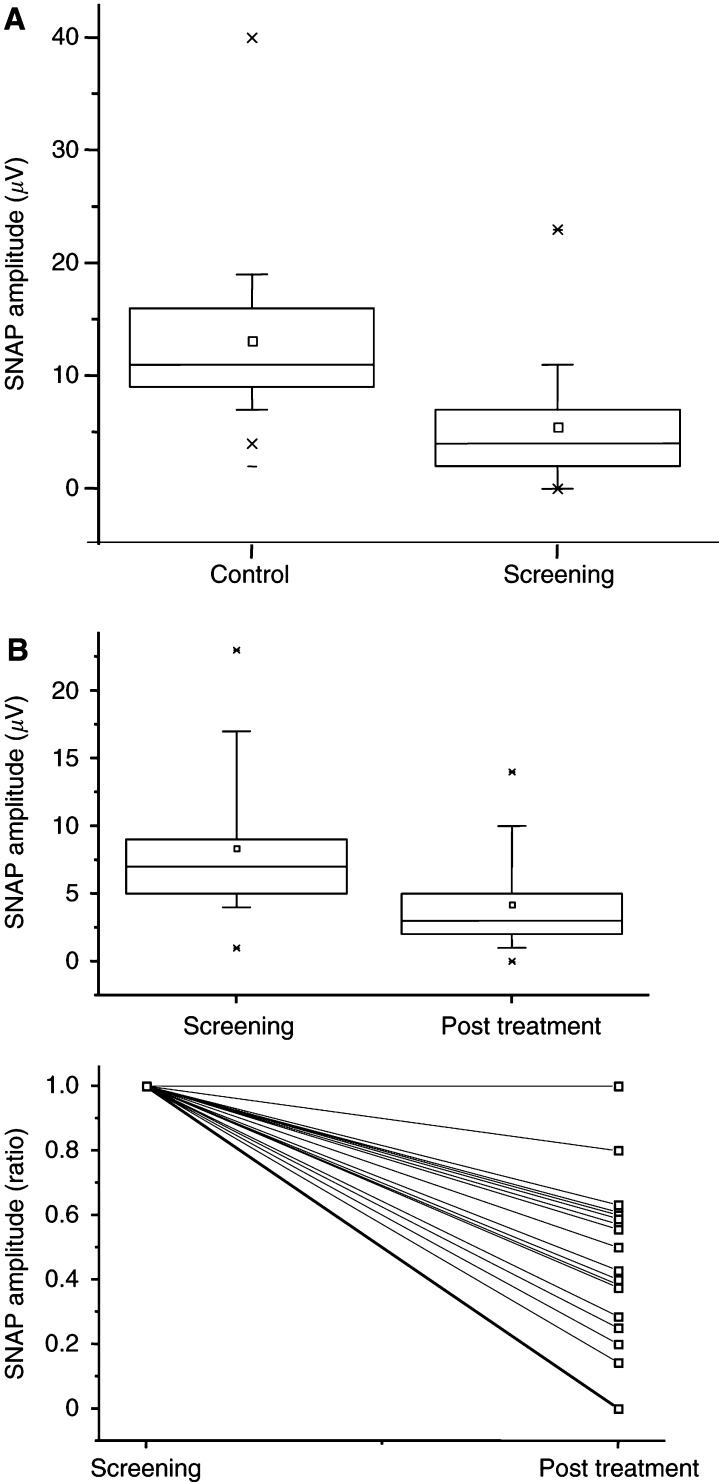
Nerve conduction study findings. Nerve conduction studies revealed a significant reduction in SNAP in men with androgen-independent prostate cancer (*n*=13) compared with reference values for men over the age of 60 (**A**). A significant fall in SNAP occurred in seven men receiving thalidomide 100 mg daily for a 6-month period (**B**).

Screening NCS was undertaken in 13 patients, nine of whom were identified as having an axonal sensory neuropathy compared with reference values for people over the age of 60 (*P*<0.01, Figure 3A

). One of these patients was a noninsulin dependent diabetic, but none of the others had an associated comorbidity or other recognised risk factor for nerve conduction deficit. None of the seven men who completed the study exhibited a symptomatic peripheral neuropathy on clinical examination at screening, but four had an axonal sensory neuropathy on NCS. At the end of the trial, all patients remained clinically asymptomatic on neurological examination, but the average of the sensory potentials showed a significant reduction, such that all seven men had an axonal sensory neuropathy (*P*=0.01, Figure 3B).

No patient with normal renal function at screening subsequently showed impairment of serum urea and electrolytes. One patient with pre-existing chronic renal failure and another with renal insufficiency because of ureteric obstruction showed further deterioration in renal function. Haematological toxicity did not occur in any patient, including one with pernicious anaemia and another with Hodgkin's disease and sarcoidosis. There was no deterioration in liver function, including in a patient with severe fatty change caused by alcoholic liver disease. One patient with painful bony metastases received intravenous strontium 4 months prior to the study and short-course external beam radiotherapy with oral corticosteroids during the study; the addition of corticosteroids did not adversely affect the clinical status of the patient.

### Quality of life

General functioning and quality of life were well maintained on thalidomide, with no significant changes for the scores on calculated scales of the SF 36 questionnaire (Table 5

**Table 5:** Short form 36 results

**Scale**	**Screening (*n*=18)**	**3 months (*n*=11)**	**6 months (*n*=6)**
Physical functioning (PF)	65.2	64.7	62.9
Social functioning (SF)	77.2	79.7	75.1
Role limitation physical (RP)	58.3	58.0	56.1
Role limitation emotional (RE)	79.0	82.3	80.8
Bodily pain (BP)	64.7	60.9	61.5
Mental health (MH)	71.2	73.4	68.2
Vitality (VT)	54.4	53.4	51.2
General health perception (GH)	56.7	57.4	54.1

). Other than the two cases of acute urinary retention documented above, there was no significant change in lower urinary tract symptoms on the ICS male questionnaire.

## DISCUSSION

The current results indicate that low-dose thalidomide can decrease PSA levels in just under 40% of patients with androgen-independent prostate adenocarcinoma, suggesting the potential for improved disease control. Six of the 16 patients for whom data were available showed a fall in PSA after starting thalidomide, which was sustained while taking the drug. Three of these completed the full study period and developed a rising PSA subsequently. A recent clinical trial of thalidomide for androgen-independent prostate cancer randomised patients to a low-dose arm of 200 mg daily, or a high-dose arm, escalating to the highest tolerated dose (up to 1200 mg) (Figg *et al*, 2001). In all, 15% of patients showed a PSA decline of over 50%, and 28% showed a decline of at least 40%, all of whom were in the low-dose arm (Figg *et al*, 2001). Higher doses appeared to result in loss of efficacy, perhaps reflecting the known immunosuppressive effects of thalidomide, which may facilitate disease progression. Our data indicate that similar effects on serum PSA are seen at an even lower dose (100 mg daily), in that 15% of patients showed a PSA decline of over 50%, with a reduced incidence of adverse effects. PSA has been proposed as a tumour marker for prostate adenocarcinoma, the levels providing an indication of disease volume and biological activity, such that a fall in PSA indicates a potential beneficial effect (Kelly *et al*, 1993; Sartor *et al*, 1998). However, it should be remembered that selection of disease markers is a problem in clinical trials for metastatic prostate cancer (Morris and Scher, 2002). As recommended by the guidelines of the Prostate-Specific Antigen Working Group (Bubley *et al*, 1999), a decline in serum PSA of at least 50% without clinical evidence of progression was employed as the main outcome measure in the current study. Nevertheless, although a decline in PSA or PSA velocity suggests clinical benefit, it is still debated how much of a decline is significant and its precise implication, while radiological assessment of metastatic disease is insensitive and difficult to interpret (Morris and Scher, 2002).

Thalidomide has been observed to increase PSA expression in a prostate cancer cell line *in vitro* (Dixon *et al*, 1999), so the PSA reduction observed in some patients probably reflects a differing effect on tumours *in vivo*. We found a significant link between the changes in PSA and circulating bFGF levels. Thalidomide is known to reduce angiogenic activity through selective inhibition of bFGF (D'Amato *et al*, 1994) and tumour-associated macrophages (Joseph and Isaacs, 1998). Since angiogenesis is important in the development and metastasis of solid tumours in general (Folkman, 1971) and it is a negative prognostic marker in prostate cancer specifically (Eckhardt and Pluda, 1997), this may reflect the mechanism by which thalidomide is acting in androgen-independent prostate cancer.

Peripheral neuropathy is a recognised complication of thalidomide (Tseng *et al*, 1996), with older persons at greater risk (Ochonisky *et al*, 1994). We demonstrated that nine out of 13 patients in this study, including four of the seven men who were on treatment for 6 months, had a ‘para-neoplastic neuropathy’ at screening, which has previously been reported in association with prostate cancer (Lucchinetti *et al*, 1998). By the end of the study, all patients tested had a sensory neuropathy on NCS, and would have had to discontinue treatment on the basis of safety recommendations in the presence of a 50% decrease in measured parameters (Gardner-Medwin *et al*, 1994). While thalidomide is a potential contributory factor, the para-neoplastic neuropathy seen in the majority of screened patients may have emerged further during the study period. Local pelvic plexus infiltration could also explain the NCS findings, but would have to be symmetric and widespread and no corroborating clinical features were detected indicating this pathology. A high incidence of peripheral neuropathy in androgen-independent prostate cancer has been reported previously in a study that also recorded onset of symptomatic peripheral neuropathy in six out of 67 patients receiving a daily dose of at least 200 mg (Molloy *et al*, 2001). In this study, six out of eight men treated for 6 months and all three men treated for 9 months developed a neuropathy. The fact that no patient developed symptoms in the current study argues in favour of lower dose treatment, which requires close clinical supervision and electrophysiological monitoring nevertheless. Future studies might usefully address use of even lower doses.

Further reported adverse effects of thalidomide include constipation, headache, nausea, weight gain, oedema, transient rashes and somnolence (Tseng *et al*, 1996). These effects are generally minor. Neither the SF 36 nor the ICS male questionnaire scores showed any significant change in the current study. Scores on the calculated SF 36 scales at screening were worse than values for healthy men of equivalent age (Brazier *et al*, 1992; Ware *et al*, 1993), or men with benign prostatic hyperplasia and hypertension (Ware *et al*, 1993). The catastrophic consequences of previous use of thalidomide to treat morning sickness of pregnancy means its use in current practice requires sensitive handling and precautions to avoid women of child-bearing potential being exposed to the drug. Thalidomide is unlicensed in the United Kingdom and only available on a named-patient basis. It was granted FDA approval in the USA in 1998, for use in cutaneous manifestations of leprosy, under controls requiring counselling and detailed consent (Zeldis *et al*, 1999).

The current results suggest that thalidomide at a dose of 100 mg daily influences the disease process in a subgroup of patients with androgen-independent prostate cancer, although no predictor of response has yet been identified. The side effect profile and the possibility of response mean that thalidomide can legitimately be discussed with patients who have failed other forms of therapy, but the development of sensory changes on NCS requires careful monitoring. Most angiogenesis-inhibiting agents are cytostatic and theoretically could provide additional benefit in combination with other chemotherapeutic agents, particularly where the overall tumour burden is low (Figg *et al*, 2001). A reduction in serum PSA of at least 50%, as seen in three men in the current study, may predict a relatively prolonged survival (Kelly *et al*, 1993; Sartor *et al*, 1998). Consequently, low-dose thalidomide may have potential as an adjunct to hormonal manipulation and other agents in therapy of poor prognosis or advanced prostate cancer, although further work is required to identify those likely to benefit.
